# An Online Monitoring Method for Low-Frequency Dielectric Loss Angle of Mining Cables

**DOI:** 10.3390/s23094273

**Published:** 2023-04-25

**Authors:** Yanwen Wang, Peng Chen, Chen Feng, Jiyuan Cao

**Affiliations:** 1 School of Mechanical, Electronic & Information Engineering, China University of Mining and Technology (Beijing), Beijing 100083, China; 2 College of Electrical Engineering and Automation, Shandong University of Science and Technology, Qingdao 266590, China

**Keywords:** dielectric loss angle, signal injection method, simultaneous measurement, mining cable, online monitoring

## Abstract

The dielectric loss angle can better reflect the overall insulation level of mining cables, so it is necessary to implement reliable and effective online monitoring of the dielectric loss angle of mining cables. In order to improve the monitoring accuracy of the dielectric loss angle tan *δ* of mining cables, a low-frequency dielectric loss angle online monitoring method combining signal injection method and double-end synchronous measurement method is proposed in this paper. Firstly, the superiority of the low-frequency signal in improving the detection accuracy of dielectric loss angle is explained, and the feasibility of the low-frequency signal injection method is analyzed. Secondly, the cable leakage is calculated using the double-terminal synchronous measurement method to measure the core current at the first and last ends of the cable, and the phase sum of the voltage at the first and last ends is selected as the reference phase quantity to realize the effective calculation of the dielectric loss angle tan *δ* of the cable. Then, the simulation model for online monitoring of dielectric loss angle of mining cable is built, and the feasibility of the online monitoring method proposed in this paper is verified by combining the simulation results. Finally, the theoretical and simulation analysis of the monitoring error of dielectric loss angle of mining cable is carried out.

## 1. Introduction

The insulation level of mining cables is directly related to the safe and stable operation of the coal mine power supply system. However, during the long-term continuous operation of the cable, its insulation performance will gradually deteriorate until insulation failure occurs, which poses a threat to the safe production of coal mines. Therefore, it is necessary to conduct online monitoring of the insulation status of mining cables [1,2].

At present, the main cable insulation online monitoring methods are the DC component method, harmonic component method, AC superposition method, DC superposition method, partial discharge method, and dielectric loss angle tangent method [3,4,5,6,7,8,9,10,11,12,13,14]. However, the DC component method and the harmonic component method are limited in their use when monitoring the insulation of cables and can only be effectively monitored when water dendrites are present in the cable insulation. The AC superposition method’s detection signal characteristics are difficult to extract and lack reliable and scientific insulation state criteria. The DC superposition method requires series resistance in the ground wire, so its operation procedure is considered unsafe. The local discharge method can only identify the local insulation deterioration of the cable, and the attenuation of the high-frequency local discharge signal is serious and not easy to collect. Compared with the above methods, the dielectric loss angle tangent method can monitor the overall aging of the cable and has better engineering application prospects.

The dielectric loss angle can better reflect the cable insulation medium moisture, deterioration, degradation or gas discharge and other insulation defects. When measuring the dielectric loss angle, the voltage and leakage current flowing through the cable are usually measured with voltage transformers and current transformers, and then the dielectric loss angle tangent value is calculated using a fast Fourier transform. At present, the effective dielectric loss angle measurement methods are the bridge balance method and 0.1 Hz VLF dielectric loss angle tester. The QS 1 type Schering bridge has become the standard for cable dielectric loss measurement due to the high accuracy of its measurements. However, it is difficult for the QS 1 type Schering bridge to meet the field measurement conditions because of the complex adjustment required during the measurement process and the uncontrollable random factors in field measurement, which affect its measurement accuracy. Sema designed the KMT VLF Sinus 0.1 Hz device to measure the dielectric loss angle of cable samples by injecting a sinusoidal voltage of 0.1 Hz [15]. However, the injected voltage of this device is 1.5 U_0_, which can easily cause secondary damage to the cable sample. It is worth noting that the above method can only test the cable offline and cannot meet the demand for real-time online monitoring.

The traditional method of measuring the dielectric loss angle tan *δ* is to collect the leakage current and the voltage to ground under the industrial frequency condition and calculate the phase of the fundamental wave of the leakage current and the voltage to ground via fast Fourier transform, and then find the dielectric loss angle. There are two main problems with this, as follows:

(1) Under the industrial frequency conditions, the value of the dielectric loss angle tan *δ* when the cable insulation is normal is particularly small, even if the insulation level of the cable decreases, the change of tan *δ* value remains small, and it is difficult to characterize the change of tan *δ* in the cable insulation level. Solving the problem of low sensitivity of the cable dielectric loss angle tangent value to the insulation level change under the industrial frequency condition is the key to online monitoring of the dielectric loss angle.
(2) Due to the existence of core resistance and residual inductance in the cable, when the load current flows into the load equipment at the end of the cable, there will also be part of the voltage on the core resistance and residual inductance, resulting in a large voltage difference between the first and last ends of the cable, and the voltage difference will change with the change in the cable load current. Therefore, when the voltage to ground at different locations on the cable is selected as the reference phase for online monitoring of the cable dielectric loss angle, the monitoring results are often different.

In recent years, in order to realize the online monitoring of the cable dielectric loss angle, many experts and scholars have proposed some methods for online monitoring of the dielectric loss angle. In the literature [16], the instantaneous value of leakage current is calculated based on the measurement of the core currents at the first and last ends of the cable, and the cable dielectric loss angle is obtained by combining the reference voltage. The literature [17] proposes a phase-to-phase dielectric loss relative variation analysis method based on the leakage current vector difference by separating the leakage currents of the cross-interconnection system. However, both methods measure the cable dielectric loss under the conditions of working frequency. Under the working frequency, the insulation dielectric loss value of the cable is small, and it is also affected by electromagnetic interference, sensor accuracy and other factors, so the cable dielectric loss value cannot be measured accurately. Therefore, related scholars have proposed measuring cable dielectric loss values under low-frequency conditions. The literature [18] proposed injecting low-frequency signals into the system through the open incremental configuration of voltage transformers (PTs) to achieve low-frequency dielectric loss measurement of cables. However, this method does not discuss the frequency of the injected signal and the effect on the detection accuracy. Based on the low-frequency dielectric loss measurement method, this paper proposes a low-frequency dielectric loss angle online monitoring method combining the signal injection method and the double-end synchronous measurement method. The detailed sections are arranged as follows: Section 2 introduces the principles and methods of the signal injection method and the double-end synchronous measurement method, and analyzes their feasibility in detail; Section 3 builds a simulation model for online monitoring of dielectric loss angle of mining cables, and the simulation results verify the feasibility of the method; Section 4 presents the theoretical and simulation analysis of the error of the method; the conclusion is given in Section 5.

## 2. Methods and Principles

### 2.1. Signal Injection Method

**Figure 1:**
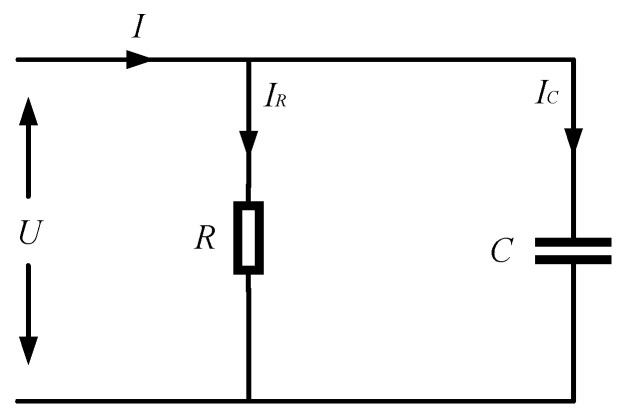
Cable Equivalent Circuits.

**Figure 2:**
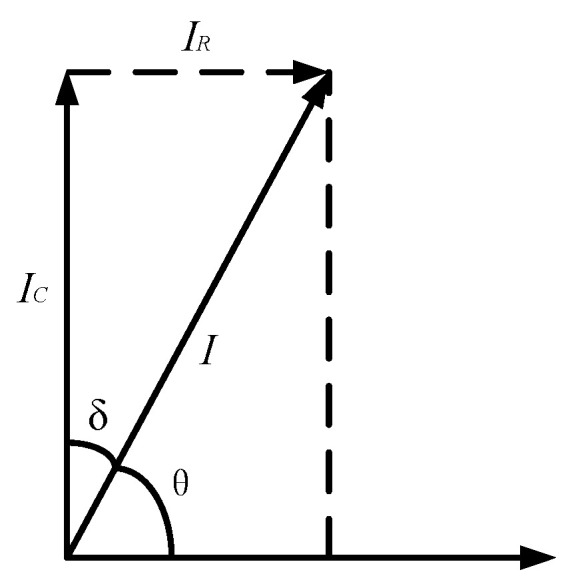
Corresponding phasor diagram.

The insulation characteristics of the cable can be equated to the RC parallel circuit model, and its equivalent circuit diagram is shown in Figure 1. *R* is the equivalent insulation resistance of the cable insulation medium, and *C* is the equivalent distributed capacitance of the cable insulation medium. The corresponding vector diagram of the model is shown in Figure 2, where *δ* is the dielectric loss angle and *θ* is the phase difference between the fundamental voltage and the fundamental current. From the phasor diagram, it can be determined that

(1) $$\tan\delta =\frac{I_{R}}{I_{C}}=\frac{1}{2\pi fCR}=\tan(90^{{}^{\circ}}-\theta )$$

where, *I_R_*, *I_C_* are the current flowing through the cable insulation medium equivalent insulation resistance and distribution capacitance, respectively, *f* is the fundamental frequency, *R* is the cable insulation medium equivalent insulation resistance and *C* is the cable insulation medium equivalent distribution capacitance.

**Figure 3:**
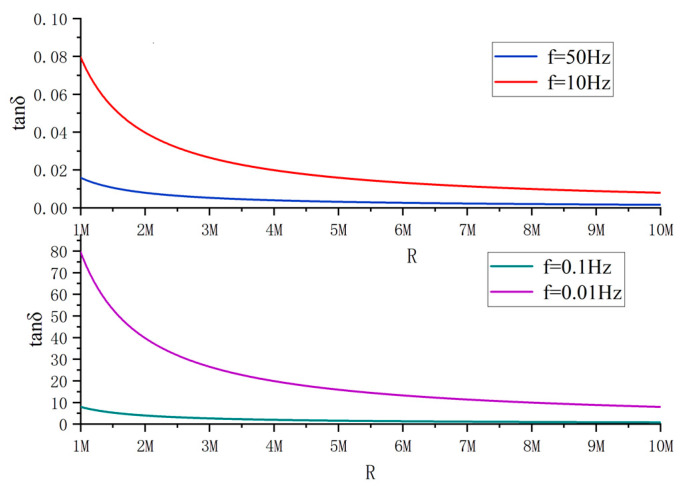
The relationship curve between tan *δ* and insulation resistance under different frequency signals.

Theoretically, the original 6 kV voltage can be used as the signal source for measuring the dielectric loss angle in the coal mine 6 kV power supply system. However, under the industrial frequency condition, it is difficult to realize the effective separation of resistive and capacitive components because the difference between resistive and capacitive components of leakage current is too large. Figure 3 shows the relationship curve between tan *δ* and insulation resistance at different signal frequencies. It can be seen that when the cable insulation level changes, the lower the signal frequency, the greater the change in the value of tan *δ*; in other words, the higher the sensitivity of the dielectric loss angle to the monitoring of the cable insulation condition. Since the reactance value of the distributed capacitor is directly related to the frequency of the injected signal, if the reactance value of the distributed capacitor can be raised to a level similar to the insulation resistance, this will undoubtedly help to improve the measurement accuracy of tan *δ*. Based on this idea, more and more researchers have focused their research on new solutions for measuring tan *δ* via low-frequency signal injection.

**Figure 4:**
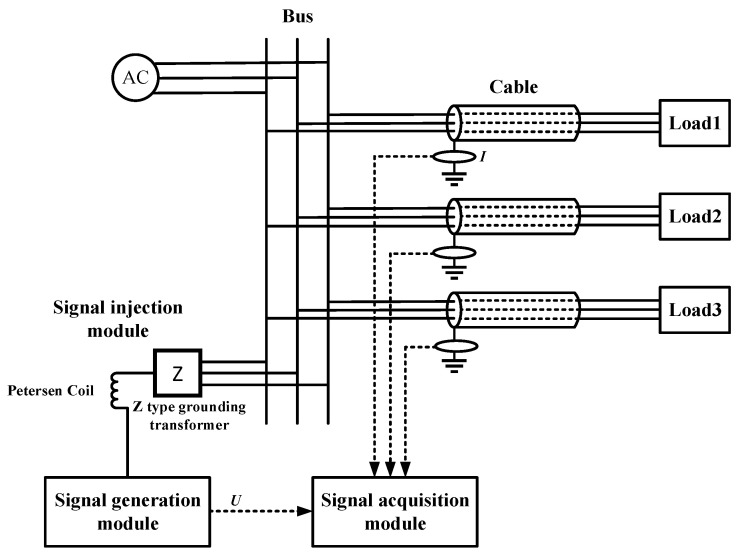
Schematic diagram of-low frequency signal injection method.

Figure 4 shows the measurement schematic of the low-frequency signal injection method. The low-frequency signal injection method has three modules: the signal generation module, the signal injection module and the signal acquisition module. The signal generation module generates sinusoidal voltage signals with adjustable frequency and amplitude, and the injected signals are injected into the 6 kV busbar of the coal mine through the signal injection module and flow into the earth through the insulation and shielding layer of the cable to form a complete circuit. Since the voltage signal applied to the three phases is the same, the injected signal can be regarded as the zero-sequence component. Thus, the dielectric loss angle tangent is calculated in the zero-sequence network.

**Figure 5:**
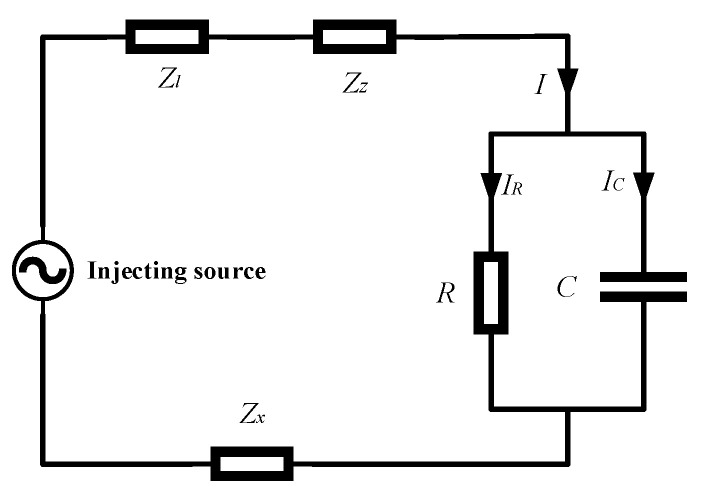
Equivalent circuit diagram of low-frequency signal injection method.

Figure 5 shows the equivalent circuit of the low-frequency signal injection method. *Z_l_*, *Z_z_* and *Z_x_* are the equivalent impedances of the arc extinguishing coil, the Z-type ground transformer and the metal shield of the cable, respectively. It is worth noting that the equivalent circuit does not have the equivalent impedance of the load, which is because the load at the end of the cable is not grounded and is equivalent to an open circuit in the whole system. When the frequency of the injected signal is particularly low, the values of *Z_l_*, *Z_z_* and *Z_x_* are small and essentially negligible compared to the equivalent insulation resistance of a cable in the milliohm range. Therefore, the capacitive and resistive components of the leakage current can be approximated as:

(2) $${\dot{I}}_{C}\approx j2\pi fC\times \dot{U}$$

(3) $${\dot{I}}_{R}\approx \frac{1}{R}\times \dot{U}$$

where ${\dot{I}}_{C}$ and ${\dot{I}}_{R}$ are the capacitive and resistive components of the leakage current $\dot{I}$, respectively. ${\dot{I}}_{R}$ and $\dot{U}$ are in the same phase, and the phase of ${\dot{I}}_{C}$ is 90 degrees ahead of $\dot{U}$. Therefore, ${\dot{I}}_{C}$ and ${\dot{I}}_{R}$ can be extracted from $\dot{I}$ based on the phase difference between $\dot{U}$ and $\dot{I}$. According to Equation (1), the value of dielectric loss angle tan *δ* can be obtained.

### 2.2. Feasibility Analysis of Low Frequency Signal Injection Method

This section will analyze the feasibility of the low-frequency signal injection method cable dielectric loss angle tan *δ* based on the following aspects.

First, the first thing that should be considered is the voltage value of the injected voltage signal. Because the principle of the signal injection method is to superimpose a low-frequency signal on the original 6 kV frequency supply network, the amplitude of the injected voltage signal must be discussed in order not to affect the normal and stable operation of the coal mine power supply system. According to the relevant provisions of IEC60038:2009 standard [19], the voltage offset of the voltage supply load at 10 kV and below should be controlled within ±7%. In this paper, the voltage amplitude of the injected signal is 180 V, which is about 3% of the industrial frequency voltage. This is lower than the national standard of voltage offset and will not affect the normal operation of the original power supply system.

Secondly, thanks to the rapid development of modern power electronics technology, the frequency conversion technology of the injected signal is widely used at the present time. At the same time, the frequency of the injected signal is low, so it is difficult for the injected signal to enter the secondary circuit through the transformer, which in turn will not affect other equipment.

Third, in order to inject the signal successfully into each line, the neutral point must be artificially created and injected into the 6 kV supply bus through the midline point, and then injected into each line from the bus. Therefore, this paper selects Z-type grounding transformer as the artificial neutral point access injection signal source. The Z-type transformer has a very large equivalent impedance in the face of positive or negative sequence signal; while in the face of zero sequence signal, the equivalent impedance is very small. Based on the principle of the low-frequency signal injection method, it is known that the dielectric loss angle tan *δ* is calculated in the zero-sequence network. The use of a Z-type grounding transformer can not only reduce the voltage division in the injected signal network and increase the voltage of the injected signal in the cable but also effectively isolate the 6 kV power supply system from the injected source and prevent the high-voltage system from entering the injected source for damage.

Fourth, most of the end loads in the 6 kV power supply system in coal mines are ungrounded loads. Therefore, in the face of the injection into the low-frequency signal, it can be equivalent to an open circuit. The current generated by the injected low-frequency signal only passes through the insulation and shielding of the cable and does not enter the load, so the method proposed in this paper will not be affected by the load, further improving the reliability of the method.

Fifth, compared with the traditional off-line detection method, the injected voltage in this paper is much smaller, and the dielectric loss angle of the cable can be monitored in real time without affecting the normal operation of the system. At the same time, the injected low-frequency signal is more helpful to improve the detection accuracy of tan *δ*. When the cable insulation drops, the rate of change of tan *δ* is greater, which is beneficial for us to judge the insulation status of the cable. In order to further improve the detection accuracy of the cable dielectric loss angle, we will use the double-end synchronous measurement method to calculate the value of the dielectric loss angle tan *δ* to ensure that the low-frequency signal injection method has strong measurement accuracy and practicability. The details of the double-end synchronous measurement method will be discussed in Section 2.3.

Finally, in order to realize the synchronous acquisition of voltage and current signals, the voltage and current signals at the first and last ends are acquired synchronously using the wide-area synchronous measurement system, which minimizes the measurement error of dielectric loss angle tan *δ* caused by non-synchronous signal acquisition and further improves the detection progress of the dielectric loss angle.

### 2.3. Double-End Synchronous Measurement Method

It can be seen from Formula (1) that monitoring the dielectric loss angle of the cable is actually monitoring the difference between the leakage current flowing through the cable insulation medium and the phase angle of the ground voltage. In other words, if the leakage current flowing through the cable insulation material and the voltage at both ends of the insulation material can be monitored, the fundamental wave of the leakage current and the voltage at both ends can be obtained via fast Fourier transform, and then the cable dielectric loss angle tangent can be calculated. It is assumed that the transmission end of the cable is defined as the first end of the cable, and the receiving end of the cable is defined as the end of the cable. Since the cable itself is passive, if the first core current and the end core current of a phase of the cable can be measured simultaneously, it is known from the current continuity theory that the current flowing through the main insulation of the cable (leakage current) is equal to the difference between the first core current and the end core current of the cable.

However, due to the cable core resistance and residual inductance, the load current in the cable will form a voltage drop across the core resistance and residual inductance of the cable, resulting in a large voltage difference between the first and last ends of the cable, and the voltage difference will change with the change in the cable load current. Therefore, when the voltage to ground at different locations on the cable is selected as the reference phase for online monitoring of the cable dielectric loss angle, the monitoring results are often different. At the same time, the monitoring results are also affected by the variation of the load current. Choosing a suitable reference phase of ground voltage and avoiding the influence of load current variation are the primary problems to be solved using the double-end synchronous measurement method.

**Figure 6:**
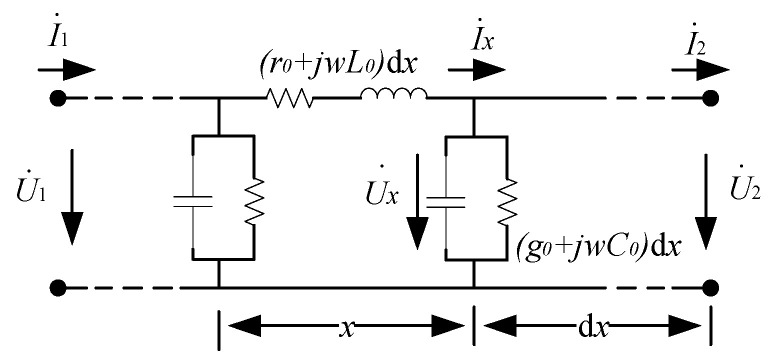
Equivalent Distributed Parameter Model of a Certain Phase of Cable.

Figure 6 shows the equivalent model of a phase distribution parameter of the cable. Let the total length of the cable be *l*; d*x* is a certain micro-element at *x* distance from the first end of the cable, *r*_0_, *L*_0_ are the resistance and inductance values of the cable core unit length and *g*_0_ and *C*_0_ are the insulation conductance and distributed capacitance values of the main insulation unit length of the cable, respectively. ${\dot{U}}_{1}$ and ${\dot{I}}_{1}$ are the voltage and current at the head of the cable line, ${\dot{U}}_{2}$ and ${\dot{I}}_{2}$ are the voltage and current at the end of the cable line, ${\dot{U}}_{x}$ and ${\dot{I}}_{x}$ are the voltage and current at the distance *x* from the head of the cable, respectively. At point *x*, based on Kirchhoff’s voltage law, we have

(4) $$d{\dot{U}}_{x}=-(r_{0}+j\omega L_{0}){\dot{I}}_{x}=-Z_{0}{\dot{I}}_{x}$$

From Kirchhoff’s current law, it follows that

(5) $$d{\dot{I}}_{x}=-(g_{0}+j\omega C_{0}){\dot{U}}_{x}=-Y_{0}{\dot{U}}_{x}$$

Simultaneous Formulas (4) and (5) can get

(6) $$\{\begin{array}{c} \frac{d^{2}{\dot{U}}_{x}}{dx^{2}}=Z_{0}Y_{0}{\dot{U}}_{x}\\ \frac{d^{2}{\dot{I}}_{x}}{dx^{2}}=Z_{0}Y_{0}{\dot{I}}_{x} \end{array}$$

Let the propagation coefficient of the cable be *γ* and the wave impedance be *Z_c_*. Its relationship with the core impedance *Z*_0_ and insulation admittance *Y*_0_ is shown in Formula (7):

(7) $$\left\{ \begin{array}{l} \gamma =\sqrt{Z_{0}Y_{0}}=\sqrt{\left(g_{0}+j\omega C_{0})(r_{0}+j\omega L_{0}\right)}\\ Z_{c}=\sqrt{\frac{Z_{0}}{Y_{0}}}=\sqrt{\frac{r_{0}+j\omega L_{0}}{g_{0}+j\omega C_{0}}} \end{array}\right.$$

Substituting Formula (7) into Formula (5), we can get:

(8) $$\{\begin{array}{c} \frac{d^{2}{\dot{U}}_{x}}{dx^{2}}=\gamma^{2}{\dot{U}}_{x}\\ \frac{d^{2}{\dot{I}}_{x}}{dx^{2}}=\gamma^{2}{\dot{I}}_{x} \end{array}$$

If the voltage at the head end of the cable and the cable are taken as known quantities to solve Equation (8), then

(9) $$\left\{ \begin{array}{l} {\dot{U}}_{x}=\frac{1}{2}({\dot{U}}_{1}+Z_{c}{\dot{I}}_{1})e^{-\gamma x}+\frac{1}{2}({\dot{U}}_{1}-Z_{c}{\dot{I}}_{1})e^{\gamma x}\\ {\dot{I}}_{x}=\frac{1}{2}(\frac{{\dot{U}}_{1}}{Z_{c}}+{\dot{I}}_{1})e^{-\gamma x}-\frac{1}{2}(\frac{{\dot{U}}_{1}}{Z_{c}}+{\dot{I}}_{1})e^{\gamma x} \end{array}\right.$$

According to the definition of hyperbolic function, Formula (9) can be rewritten as:

(10) $$\left\{ \begin{array}{l} {\dot{U}}_{x}={\dot{U}}_{1}\cosh(\gamma x)-Z_{c}{\dot{I}}_{1}\sinh(\gamma x)\\ {\dot{I}}_{x}={\dot{I}}_{1}\cosh(\gamma x)-\frac{{\dot{U}}_{1}}{Z_{c}}\sinh(\gamma x) \end{array}\right.$$

When *x* = *l*, the voltage and current at the end of the cable can be expressed in terms of the voltage and current at the first end of the cable, as shown in Equation (11):

(11) $$\left\{ \begin{array}{l} {\dot{U}}_{2}={\dot{U}}_{1}\cosh(\gamma l)-Z_{c}{\dot{I}}_{1}\sinh(\gamma l)\\ {\dot{I}}_{2}={\dot{I}}_{1}\cosh(\gamma l)-\frac{{\dot{U}}_{1}}{Z_{c}}\sinh(\gamma l) \end{array}\right.$$

Therefore, the voltage and current at the first end of the cable can be used to express the voltage difference between the first and last ends of the cable, as shown in Equation (12):

(12) $$\Delta \dot{U}={\dot{U}}_{1}-{\dot{U}}_{2}=-2{\dot{U}}_{1}\sinh^{2}(\frac{\gamma l}{2})+Z_{c}{\dot{I}}_{1}\sinh(\gamma l)$$

Similarly, if the voltage and current at the end of the cable are taken as known quantities to solve Equation (8), we can obtain:

(13) $$\left\{ \begin{array}{l} {\dot{U}}_{x}={\dot{U}}_{2}\cosh[\gamma (l-x)]+Z_{c}{\dot{I}}_{2}\sinh[\gamma (l-x)]\\ {\dot{I}}_{x}={\dot{I}}_{2}\cosh[\gamma (l-x)]+\frac{{\dot{U}}_{2}}{Z_{c}}\sinh[\gamma (l-x)] \end{array}\right.$$

When *x* = 0, the voltage and current at the head end of the cable can be expressed by the voltage and current at the end of the cable, as shown in Formula (14):

(14) $$\left\{ \begin{array}{l} {\dot{U}}_{1}={\dot{U}}_{2}\cosh(\gamma l)+Z_{c}{\dot{I}}_{2}\sinh(\gamma l)\\ {\dot{I}}_{1}={\dot{I}}_{2}\cosh(\gamma l)+\frac{{\dot{U}}_{2}}{Z_{c}}\sinh(\gamma l) \end{array}\right.$$

The voltage difference between the first and last ends of the cable can be obtained by expressing the voltage and current at the end of the cable, as shown in Equation (15):

(15) $$\Delta \dot{U}={\dot{U}}_{1}-{\dot{U}}_{2}=2{\dot{U}}_{2}\sinh^{2}(\frac{\gamma l}{2})+Z_{c}{\dot{I}}_{2}\sinh(\gamma l)$$

Subtract Formula (12) from Formula (15), and obtain:

(16) $$\frac{{\dot{I}}_{1}-{\dot{I}}_{2}}{{\dot{U}}_{1}+{\dot{U}}_{2}}=\frac{2\sinh^{2}(\frac{\gamma l}{2})}{Z_{c}\sinh(\gamma l)}$$

It can be seen from Formula (16) that the ratio of the difference between the current phasors at the first and last ends of the cable to the sum of the voltage phasors at the first and last ends of the cable is only related to the inherent parameters of the cable (propagation coefficient, wave impedance, length, etc.), independent of the load current flowing through the cable. Therefore, when conducting online monitoring of the dielectric loss angle of the cable, the sum of the voltage phasors at the first and last ends of the cable should be used as the reference phasor, so that the measurement results will not be affected by the change of the cable load current. According to Formula (16), we can obtain:

(17) $$\frac{I∠\varphi }{U∠\theta }=\frac{{\dot{I}}_{1}-{\dot{I}}_{2}}{{\dot{U}}_{1}+{\dot{U}}_{2}}$$

where *I* and *φ* are the amplitude and phase angle of the difference between the phase amounts of the first and last currents, respectively; *U* and *θ* are the amplitude and phase angle of the sum of the phase amounts of the first and last voltages, respectively. Therefore, the cable dielectric loss angle tangent can be expressed as:

(18) $$\tan\delta =\tan[90^{{}^{\circ}}-(\varphi -\theta )]$$

**Figure 7:**
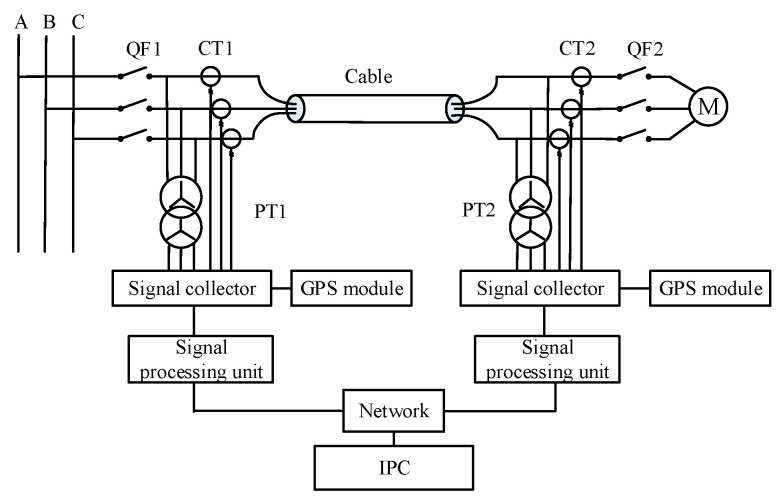
Measuring principle diagram of double-ended synchronous measurement method.

Figure 7 shows the measurement schematic of the double-end synchronous measurement method. The voltage and cable at the first and last ends of the cable are collected synchronously by the Wide Area Measurement System (WAMS). The WAMS consists of a global positioning system (GPS), a phase measurement unit (PMU), a communication network and a control center. The GPS is used for the first and last synchronous acquisition, and its role is to time the measurement equipment.

## 3. Simulation Analysis

### 3.1. Simulation Model Building

**Figure 8:**
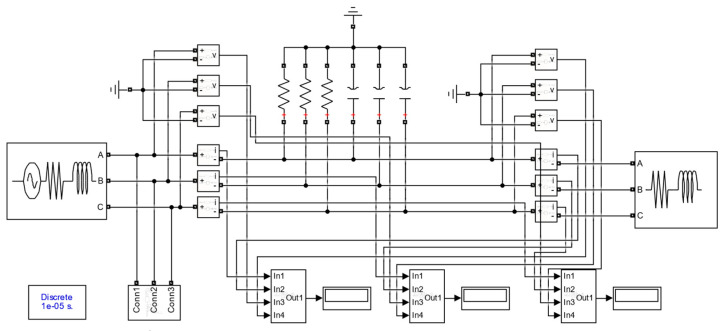
Simulation model for online monitoring of dielectric loss angle of mining cable.

In order to verify the feasibility of the online monitoring method of the dielectric loss angle of mining cable proposed in this paper, a simulation model of online monitoring of the dielectric loss angle of a mining cable is built using MATLAB, as shown in Figure 8. For the simplicity of the simulation model, the signal injection module and the dielectric loss angle tan *δ* calculation module are modularly encapsulated. In the simulation, the three-phase power supply is 6 kV, the sampling frequency is 1 kHz, the sampling duration is 1 s and the cable length is 1 km. According to the literature [18] and several simulation tests, combined with the measurement frequency range of the transformer, the low-frequency signal frequency injected in this paper is 10 Hz. Take the MYJV22-6-3 × 120 type mining 6 kV high-voltage cable as an example. With the normal cable insulation level, the factory parameters of this type of cable are as follows: *r*_0_ = 0.1959 mΩ/km, *l*_0_ = 0.3613 mH/km, *g*_0_ = 3 × 10^−8^ S/km, *C*_0_ = 0.3266 μF/km. The voltage and current at the first and last ends of the cable are measured and converted into vector form using the Fourier transform module. The cable dielectric loss angle tangent can be calculated by combining Equations (17) and (18).

**Figure 9:**
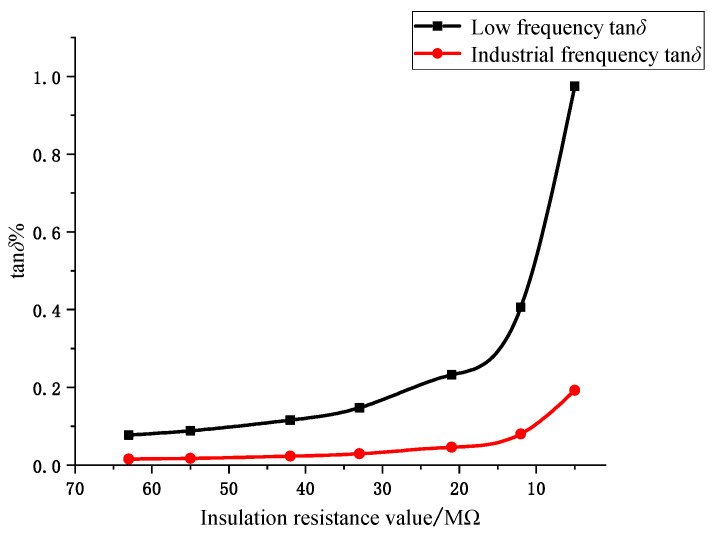
Comparison of tan *δ* variation between industrial frequency and low frequency.

In order to reflect the advantages of online monitoring of dielectric loss angle using the low-frequency signal injection method, the dielectric loss angle tan *δ* of the single-phase insulation fault of the cable is measured at low frequency and industrial frequency, respectively. By changing the resistance value of the main insulation of the cable to simulate the operating condition of the cable with different aging degrees, the change in the dielectric loss angle tan *δ* when the insulation resistance decreases from 63 MΩ to 5 MΩ is calculated. The simulation results are shown in Figure 9. It can be seen from Figure 9 that although both industrial-frequency tan *δ* and low-frequency tan *δ* can reflect the aging of cable insulation materials, at the early stage of insulation aging, low-frequency tan *δ* undergoes certain changes, while industrial-frequency tan *δ* can hardly judge the aging condition of cable insulation; at the later stage of insulation aging, low-frequency tan *δ* can reflect the changes in the aging condition of insulation materials more sensitively, compared with industrial-frequency tan *δ*, and low-frequency tan *δ* online monitoring can better reflect the insulation level of the cable.

At the same time, in order to analyze the effect of end load on the cable dielectric loss angle tan *δ* value, for the convenience of analysis, the end load in this paper will be set as the resistive load in the simulation model, and the load current will be changed by changing the magnitude of load resistance. Taking phase A as an example, the voltage phasor at the first end of the cable, the voltage phasor at the last end and the sum of voltage phasor at both ends are taken as the reference phase, and the tan *δ* value obtained via simulation calculation is shown in Table 1. In Table 1, tan *δ*_1_, tan *δ*_2_ and tan *δ*_3_ are the measured values obtained by taking the voltage phasor at the first end of the cable, the voltage phasor at the last end and the sum of voltage phasor at both ends as the reference phase quantity, respectively. Therefore, the phasor sum of the voltage at both ends is chosen as the reference phasor to calculate the tan *δ* value without the influence of load current.

### 3.2. Dielectric Loss Angle Simulation under Different Aging Conditions

When the cables are in different aging states, their dielectric loss angle tan *δ* values will change accordingly. In order to investigate the influence of different aging conditions of the cable on the value of the dielectric loss angle tan *δ*, the cable simulation model is set as follows:

(1) Set the equivalent insulation resistance of cable phase A to 100 MΩ and the equivalent distributed capacitance to 340 nF to characterize the slight aging of the insulation of cable phase A.
(2) Set the equivalent insulation resistance of phase B of the cable to 50 MΩ and the equivalent distributed capacitance to 360 nF to characterize the moderate aging of the insulation of phase B of the cable.
(3) Set the equivalent insulation resistance of the phase C of the cable to 10 MΩ and the equivalent distributed capacitance to 380 nF to characterize the severe aging of the C phase insulation of the cable.
(4) Set the equivalent insulation resistance of phase A of the cable to 1 MΩ and the equivalent distributed capacitance to 400 nF to characterize the insulation failure of phase A of the cable.
(5) Set the equivalent insulation resistance of phase A to 30 MΩ and the equivalent distributed capacitance to 370 nF, set the equivalent insulation resistance of phase B to 5 MΩ and the equivalent distributed capacitance to 390 nF and set phase C to the normal value of the cable insulation to characterize the simultaneous failure of the cable insulation of phases A and B.
(6) Set the equivalent insulation resistance of phase A to 20 MΩ and the equivalent distributed capacitance to 375 nF, set the equivalent insulation resistance of phase B to 10 MΩ and the equivalent distributed capacitance to 380 nF and set the equivalent insulation resistance of phase C to 1 MΩ and the equivalent distributed capacitance to 400 nF to characterize the simultaneous failure of the cable insulation of phases A, B and C.

Simulation of the cable dielectric loss angle tan *δ* values under different aging states; the simulation results are shown in the table. From the simulation results in Table 2, it can be seen that when the cable insulation fails, the tan *δ* value of the faulty phase increases significantly, and the cable insulation condition can be judged by the change in the tan *δ* value. The online monitoring method of dielectric loss angle of mining cable proposed in this paper can accurately reflect the insulation level of the cable under different aging conditions. Whether it is a single-phase insulation fault or a multi-phase insulation fault, it is feasible to use the double-ended synchronous measurement method to monitor the cable’s dielectric loss angle tan *δ* value online.

### 3.3. Dielectric Loss Angle Simulation under Water Tree Aging

**Figure 10:**
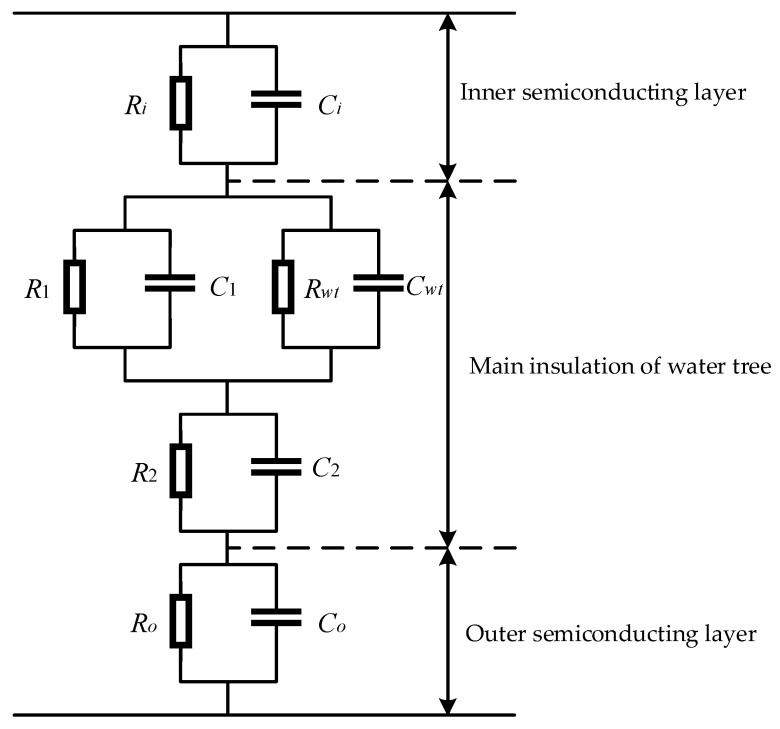
Cable water tree aging simulation model.

A simulation model of cable water tree aging was built according to the literature [20]. As shown in Figure 10, *R_in_* and *C_in_* are the equivalent insulation resistance and distributed capacitance of the inner semiconducting layer, *R_out_* and *C_out_* are the equivalent insulation resistance and distributed capacitance of the outer semiconducting layer, *R_wt_* and *C_wt_* are the equivalent insulation resistance and distributed capacitance of the water tree aging section, *R*_1_ and *C*_1_ are the equivalent insulation resistance and distributed capacitance of the water tree aging region, *R*_1_ and *C*_1_ are the equivalent insulation resistance and distributed capacitance of the parallel cable section and *R*_2_ and *C*_2_ are the equivalent insulation resistance and distributed capacitance of the series cable section in the water tree aging area. Since the equivalent impedance of the inner and outer semiconductor layers is much smaller than the equivalent impedance of the main insulation layer, it can be neglected in the simulation model.

In the existing research, the 3D cable model with 100–500 μm long spherical water tree of the cable was simulated using COMSOL finite element simulation software. The simulation results show that the equivalent insulation resistance of the cable is inversely proportional to the length of the water tree, and the equivalent distributed capacitance of the cable is proportional to the length of the water tree. Referring to the simulation results in the literature [20], the cable parameters for different lengths of water tree models are set in MATLAB, as shown in Table 3, and the parameters in the table are the parameter values per unit length.

**Figure 11:**
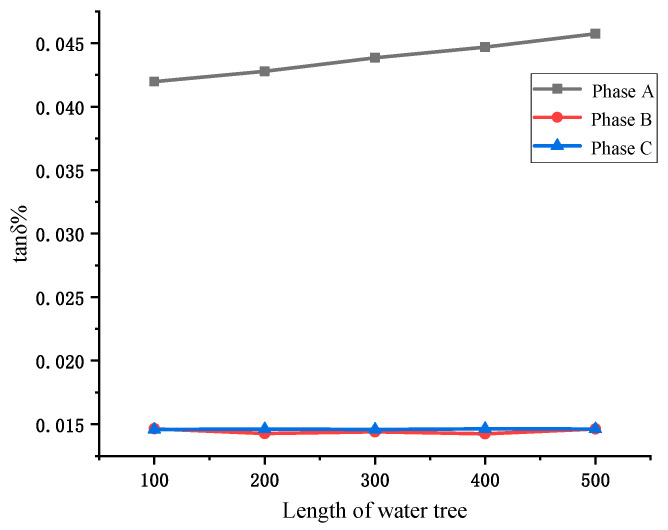
Cable dielectric loss angle tan *δ* value under different water tree length.

The simulation results of the cable dielectric loss angle tan *δ* for different water tree lengths are shown in Figure 11. From Figure 11, it can be seen that the tan *δ* value of the aging phase of the water tree aging varies with the growth length of the water tree, and the tan *δ* value of the non-aging phase remains basically the same. Therefore, the monitoring of cable water tree aging can be realized by monitoring the dielectric loss angle tan *δ*.

## 4. Error Analysis

### 4.1. Effect of Grid Harmonics

Harmonics are generated in all aspects of power generation, transmission and consumption in the coal mine grid. Electronic excitation devices such as IGBTs and SCRs can cause harmonics to be generated in generators. Since the working flux density of the transformer core is generally selected in the near-saturation region of the magnetization characteristic curve, the transformer will also cause harmonics. Likewise, non-linear power loads, such as inverters, rectifiers, inverters, etc., cause harmonic currents to flow through the grid. Therefore, it is necessary to analyze the effect of grid harmonics on the monitoring of cable dielectric loss angles.

**Figure 12:**
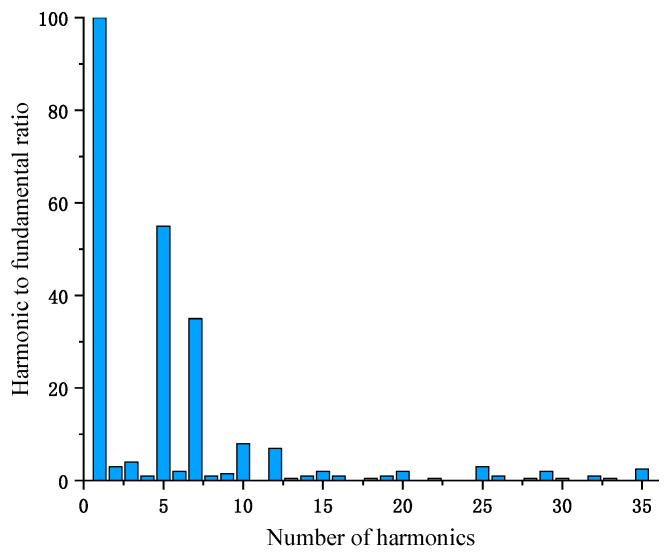
Harmonic spectrum of coal mine power grid.

In order to make the simulation closer to the actual situation, this paper analyzes the harmonic content of the coal mine grid by means of a grid power quality analyzer. Figure 12 shows the harmonic spectrum of the coal mine power grid collected by the power quality analyzer. From Figure 12, it can be seen that the main harmonic contents in the coal mine grid are the fifth and seventh harmonics.

The fifth and seventh harmonics are added to the simulation model to simulate the cable dielectric loss angle tan *δ* values. The simulation results show that the harmonics of the coal mine grid do not affect the calculation results of the tan *δ* values of the double-ended synchronous measurement method, so the influence of the grid harmonics can be ignored in practical applications.

### 4.2. Effect of Frequency Fluctuations of the Injected Signal

**Figure 13:**
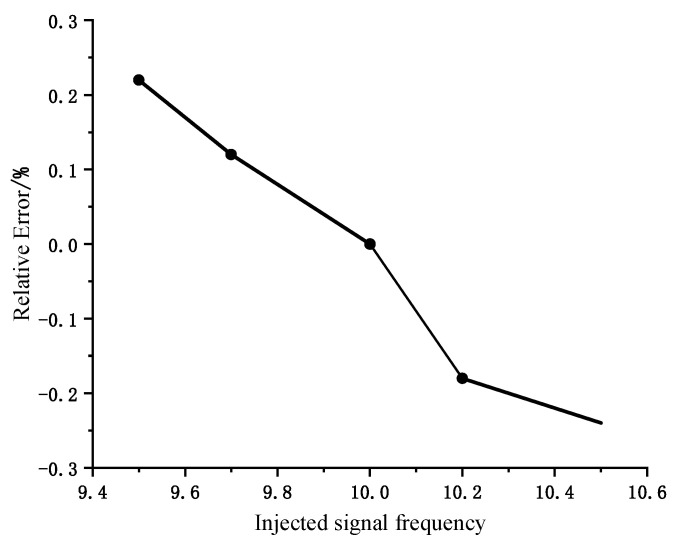
Relative error of the injected signal frequency fluctuation on tan *δ*.

In order to quantitatively analyze the effect of frequency fluctuation on the dielectric loss angle tan *δ*, by changing the injection signal frequency in the simulation model and setting the injection signal frequency to vary in the range of 9.5~10.5 Hz, the change in the tan *δ* relative error is shown in Figure 13. From Figure 13, it can be seen that when the injection signal frequency fluctuates, the tan *δ* relative error does not exceed 0.25%, and the effect on monitoring the cable insulation tan *δ* value is small.

## 5. Conclusions

The dielectric loss angle can better reflect the insulation state of the cable, as well as the growth of the water tree, so it is necessary to monitor the dielectric loss angle of mining cables online. In this paper, a low-frequency dielectric loss angle online monitoring method combining signal injection method and double-end synchronous measurement method is proposed. Firstly, the monitoring principle of the low-frequency signal injection method is explained, and the advantages of dielectric loss angle measurement under low-frequency conditions are analyzed. Secondly, the double-end synchronous measurement method is used to measure and calculate the dielectric loss angle to improve the detection accuracy of the dielectric loss angle; then, the online monitoring method proposed in this paper is simulated and verified by Matlab. Finally, the error analysis of this monitoring method is combined with the theoretical analysis and simulation results. The main conclusions are as follows:

(1) Through the cable tan *δ* values under different aging conditions, it can be found that the proposed method in this paper can accurately reflect the insulation level of the cable. When the aging phenomenon occurs in the cable insulation, the dielectric loss angle tan *δ* of the aging phase will change with the aging degree, and the deeper the aging degree, the larger the tan *δ* value.
(2) When using the double-end synchronous measurement method for tan *δ* calculation, the sum of the voltage phasors at the first and last ends is selected as the reference phase, the tan *δ* value is not affected by the load current, and the calculation is more accurate.
(3) By building the cable water tree aging model and simulating the dielectric loss angle tan *δ* values under different lengths of water trees, the dielectric loss angle of the aging phase where water tree aging occurs increases with the increase in the water tree length, and there is almost no change in the non-aging phase.
(4) Grid harmonics do not affect the measurement of tan *δ* value. When the frequency of the injected signal fluctuates in the range of 9.5~10.5 Hz, the relative error of tan *δ* measurement does not exceed 0.25%, which has less influence on the monitoring of cable insulation tan *δ* value.

## Figures and Tables

**Table 1:** Effect of different reference voltages on tan *δ*.

Load Resistance/Ω	tan *δ*_1_/%	tan *δ*_2_/%	tan *δ*_3_/%
10	0.45872	0.38267	0.01463
50	0.14726	0.08669	0.01463
100	0.09285	0.03745	0.01463
200	0.05808	0.01896	0.01463
300	0.03651	0.00982	0.01463

**Table 2:** Cable tan *δ* simulation results for different aging states.

Measurement Phase	tan *δ*_A_/%	tan *δ*_B_/%	tan *δ*_C_/%
Normal insulation	0.01463	0.01463	0.01463
Slight aging of phase A	0.04679	0.01463	0.01463
Moderate aging of phase B	0.01463	0.08838	0.01463
Severe aging of phase C	0.01463	0.01463	0.4186
Insulation fault of phase A	3.9769	0.01463	0.01463
AB two-phase insulation fault	0.1433	0.8158	0.01463
ABC three-phase insulation fault	0.2121	0.4186	3.9769

**Table 3:** Estimates of cable water tree aging parameters at different lengths.

Length/μm	*R_wt_*/MΩ	*C_wt_*/fF	*R*_1_/Ω	*C*_1_/pF	*R*_2_/Ω	*C*_2_/pF
100	0.48	0.11	8.14 × 10^9^	2.50	3.58 × 10^10^	202.50
200	0.24	0.24	4.07 × 10^9^	5.00	1.79 × 10^10^	405.00
300	0.16	0.37	2.72 × 10^9^	7.50	1.19 × 10^10^	607.50
400	0.12	0.44	2.14 × 10^9^	9.51	0.94 × 10^10^	810.00
500	0.10	0.55	1.63 × 10^9^	12.49	0.72 × 10^10^	1012.50

## Data Availability

Not applicable.
